# Clinical features of lenvatinib for unresectable hepatocellular carcinoma in real‐world conditions: Multicenter analysis

**DOI:** 10.1002/cam4.1909

**Published:** 2018-12-21

**Authors:** Atsushi Hiraoka, Takashi Kumada, Kazuya Kariyama, Koichi Takaguchi, Masanori Atsukawa, Ei Itobayashi, Kunihiko Tsuji, Kazuto Tajiri, Masashi Hirooka, Noritomo Shimada, Hiroshi Shibata, Toru Ishikawa, Hironori Ochi, Toshifumi Tada, Hidenori Toyoda, Kazuhiro Nouso, Akemi Tsutsui, Norio Itokawa, Michitaka Imai, Kouji Joko, Yoichi Hiasa, Kojiro Michitaka, the Real‐life Practice Experts for HCC (RELPEC) Study Group, HCC 48 Group (hepatocellular carcinoma experts from 48 clinics in Japan)

**Affiliations:** ^1^ Gastroenterology Center Ehime Prefectural Central Hospital Matsuyama Japan; ^2^ Department of Gastroenterology and Hepatology Ogaki Municipal Hospital Gifu Japan; ^3^ Department of Gastroenterology Okayama City Hospital Okayama Japan; ^4^ Department of Hepatology Kagawa Prefectural Central Hospital Takamatsu Japan; ^5^ Division of Gastroenterology and Hepatology, Department of Internal Medicine Nippon Medical School Tokyo Japan; ^6^ Department of Gastroenterology Asahi General Hospital Asahi Japan; ^7^ Center of Gastroenterology Teine Keijinkai Hospital Sapporo Japan; ^8^ Department of Gastroenterology Toyama University Hospital Toyama Japan; ^9^ Department of Gastroenterology and Metabology Ehime University Graduate School of Medicine Toon Japan; ^10^ Division of Gastroenterology and Hepatology Otakanomori Hospital Kashiwa Japan; ^11^ Department of Gastroenterology Tokushima Prefectural Central Hospital Tokushima Japan; ^12^ Department of Gastroenterology Saiseikai Niigata Daini Hospital Niigata Japan; ^13^ Hepato‐biliary Center Matsuyama Red Cross Hospital Matsuyama Japan

**Keywords:** adverse event, albumin‐bilirubin grade, alpha‐fetoprotein, hand‐foot skin reaction, hepatocellular carcinoma, lenvatinib, muscle volume, regorafenib, sorafenib

## Abstract

**Background/Aim:**

Presently, there are no therapeutic options for unresectable hepatocellular carcinoma (u‐HCC) patients who are intolerant to sorafenib or regorafenib failure. There have been no reports with detailed clinical findings of lenvatinib (LEN), a newly developed first‐line tyrosine kinase inhibitor (TKI), obtained in real‐world practice. We aimed to elucidate the therapeutic efficacy of LEN.

**Materials/Methods:**

From March to August 2018, 105 u‐HCC patients were treated with LEN. Following exclusion of those who started with a reduced LEN dose and/or had a short observation period (<2 weeks), 77 patients (72.0 ± 8.9 years, 59 males, 8 mg/12 mg = 49/28, Liver Cancer Study Group of Japan 6th [LCSGJ]‐TNM stage II/III/IVa/IVb = 8/28/4/37, and American Joint Committee on Cancer/Union for International Cancer Control 8th [AJCC/UICC]‐TNM stage IB:II:IIIA:IIIB:IVA:IVB = 2:27:6:5:9:28) were divided into two groups (TKI naïve [n = 33] and TKI experienced [n = 44], including 11 with regorafenib history). Therapeutic response was evaluated using mRECIST. Clinical data were retrospectively evaluated.

**Results:**

There were significant differences in age (74.6 ± 11.2 vs 70.0 ± 5.9 years, *P* = 0.040), LCSGJ‐TNM (II:III:IVa:IVb = 8:12:1:12 vs 0:16:3:25, *P* = 0.006), and AJCC/UICC‐TNM (IB:II:IIIA:IIIB:IVA:IVB = 2:17:1:1:4:8 vs 0:10:5:4:5:20, *P* = 0.028), while hepatic reserve function, adverse event (AE) profiles, and progression‐free survival (89.7%/80.4% vs 90.5%/80.1%, *P* = 0.499) and overall survival (96.7%/96.7% vs 100%/92.3%, *P* = 0.769) after 4 and 12 weeks were not significantly different between the TKI‐naïve and TKI‐experienced groups. Overall response rate and disease control rate at 4 weeks (n = 52) were 38.5% and 80.8%, respectively, and 32.4% and 70.3%, respectively, at 12 weeks (n = 37). A significant decline in log10 AFP from the baseline to 4 weeks after introducing LEN was observed in patients with PR and SD (2.047 ± 1.148 vs 1.796 ± 1.179, *P* < 0.001).

**Conclusion:**

Regardless of past TKI therapy, therapeutic response and AEs after introducing LEN were similar. LEN may be an important treatment for the present unmet need regarding TKI treatment against u‐HCC.

## 1 INTRODUCTION

For treatment of unresectable hepatocellular carcinoma (u‐HCC), tyrosine kinase inhibitors (TKIs), such as sorafenib (SOR)^1^, ^2^ and regorafenib (REG),^3^, ^4^ have been introduced. SOR was developed as a first‐line agent for u‐HCC, while REG is used as second‐line therapy in patients who show good tolerability and progressive disease (PD) with SOR. Recently, lenvatinib (LEN), a new TKI,^5^, ^6^ has become available as a first‐line drug for u‐HCC. However, though the number of TKIs for u‐HCC has increased, there is presently no therapeutic option for patients with SOR failure or who show intolerability for SOR as well as failure with REG, indicating an important and practical unmet need. Following the introduction of LEN in clinical practice in Japan in March 2018, the drug has been used in real‐world practice not only for TKI‐naïve but also for TKI‐experienced patients as second‐ or third‐line treatment.^7^ On the other hand, therapeutic response and adverse events (AEs) associated with LEN treatment for u‐HCC patients with and without a past history of TKI have not been elucidated. In the present study, we analyzed clinical features of patients who received LEN treatment for u‐HCC for therapeutic response and AEs.

## 2 MATERIALS AND METHODS

**Figure 1:**
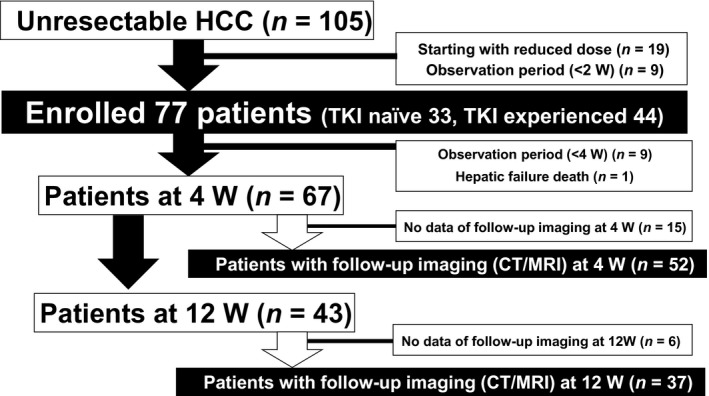
Flow diagram of enrolled patients. HCC, hepatocellular carcinoma; TKI, tyrosine kinase inhibitor; W, weeks

From March to August 2018, LEN (Lenvima^®^, Eisai Co., Ltd., Tokyo, Japan) was given to 105 u‐HCC Japanese patients receiving treatment at 13 different institutions in Japan (Ehime Prefectural Central Hospital [n = 19], Okayama City Hospital [n = 12], Kagawa Prefectural Central Hospital [n = 12], Nippon Medical School Hospital [n = 12], Asahi General Hospital [n = 10], Teine Keijinkai Hospital [n = 7], Ogaki Municipal Hospital [n = 6], Toyama University Hospital [n = 6], Ehime University Hospital [n = 6], Otakanomori Hospital [n = 5], Tokushima Prefectural Central Hospital [n = 4], Saiseikai Niigata Daini Hospital [n = 3], Matsuyama Red Cross Hospital n = 3]). We retrospectively examined the records of those patients and collected clinical data obtained at the introduction of LEN, as well as after 2 weeks and every 4 weeks thereafter. Following exclusion of those who started with a reduced LEN dose and/or had a short observation period (<2 weeks), 77 patients were enrolled and divided into two groups, TKI naïve (n = 33) and TKI experienced (n = 44), according to their past history with TKI treatments. Flow diagram of enrolled patients is shown in Figure 1. Clinical characteristics, therapeutic response including progression‐free survival rate (PFSR) and overall survival rate (OSR), and AEs were analyzed in a retrospective manner.

Patients positive for anti‐hepatitis C virus (HCV) were judged to have HCC due to HCV, while those positive for hepatitis B virus surface antigen (HBsAg) were judged to have HCC due to hepatitis B virus (HBV).

### 2.1 Assessment of hepatic reserve function and prognosis.

Child‐Pugh classification^8^ and ALBI grade were used to assess hepatic reserve function. ALBI grade was calculated based on serum albumin and total bilirubin values using the following formula: [ALBI score = (log10 bilirubin (µmol/L) × 0.66) + (albumin (g/L) × −0.085)], and defined by the following scores: ≤−2.60 = Grade 1,>−2.60 to ≤−1.39 = Grade 2,>−1.39 = Grade 3.^9^, ^10^ For a more detailed evaluation of the middle grade of ALBI (grade 2), we used a modified ALBI (mALBI) grade by creating 4 grades, including sub‐grading for the middle grade (2a and 2b), with an ALBI score of −2.270, which was reported as the cutoff value for indocyanine green retention 15 minutes 30%, used as the value for dividing 2a and 2b.^12^, ^13^

### 2.2 Assessment of muscle volume

Muscle volume loss (MVL) was determined using the following previously reported index: [psoas index (PI): bilateral psoas muscle area of middle L3 level (cm^2^)/height (m)^2^; cutoff values for MVL of males = 4.24 cm^2^/m^2^, females = 2.50 cm^2^/m^2^], based on CT findings.^14^ PI was manually calculated using psoas muscle area at the middle L3 level in CT findings with personal computer software (Centricity Web DX ver.3.7.3.6417: GE Healthcare Japan, Tokyo, Japan, or OsiriX DICOM Viewer MD 9.5: https://www.osirix-viewer.com). Muscle volume was calculated using computed tomography (CT) findings obtained at the start of LEN and again after 4 and 12 weeks in patients for whom CT imaging data were available.

### 2.3 Diagnosis and treatment of HCC

HCC was diagnosed based on an increasing course of alpha‐fetoprotein (AFP), as well as dynamic CT,^15^ magnetic resonance imaging (MRI),^16^, ^17^ contrast‐enhanced ultrasonography (CEUS) with perflubutane (Sonazoid^®^, Daiichi Sankyo Co., Ltd. Tokyo, Japan),^18^, ^19^ and/or pathological findings. American Joint Committee on Cancer (AJCC)/Union for International Cancer Control (UICC) tumor node metastasis (TNM) stage was used for evaluation of tumor progression as well as TNM stage, which was determined as previously reported in studies for staging of HCC conducted by the Liver Cancer Study Group of Japan (LCSGJ).^20^ The present study protocol was approved by the Institutional Ethics Committee of Ehime Prefectural Central Hospital (No. 29‐75).

### 2.4 LEN treatment and assessment of AEs

After obtaining written informed consent, LEN was orally administered at 8 mg/d to patients weighing <60 kg and 12 mg/d to those ≥60 kg, and discontinued when any unacceptable or serious AE or clinical tumor progression was observed. According to the guidelines for administration of LEN, the drug dose should be reduced or treatment interrupted when a patient develops any grade 3 or more severe AE or if any unacceptable grade 2 drug‐related AE occurs. AEs were assessed according to the National Cancer Institute Common Terminology Criteria for Adverse Events, version 4.0.^21^ The worst grade for each AE during the present observation period was recorded. If a drug‐related AE occurred, dose reduction or temporary interruption was maintained until the symptom was resolved to grade 1 or 2, according to the guidelines provided by the manufacturer.

### 2.5 Evaluation of therapeutic response

Local physicians at each institution evaluated tumors using enhanced CT or MRI results obtained at 4, 8, or 12 weeks after introducing LEN, in accordance with the modified RECIST guidelines.^22^, ^23^

### 2.6 Statistical analysis

Data are expressed as the mean and standard deviation (SD). Statistical analyses were performed using Welch's *t* test, Fischer's exact test, Mann‐Whitney's *U* test, a paired *t* test, Wilcoxon signed‐rank test, Kaplan‐Meyer method, and a log‐rank test. A *P* value less than 0.05 was considered to indicate statistical significance. All statistical analyses were performed using Easy R (EZR), version 1.29 (Saitama Medical Center, Jichi Medical University, Saitama, Japan),^24^ a graphical user interface for R (The R Foundation for Statistical Computing, Vienna, Austria).

## 3 RESULTS

Following exclusion of patients whose initial LEN dose was reduced or those with a short observation period <2 weeks, a total of 77 were analyzed and their characteristics are shown in Table 1. The average observation period for all subjects after introduction of LEN was 79.3 ± 40.7 days. Forty‐four (57.1%) had a past history of SOR treatment (TKI‐experienced group), including 11 (14.4%) who were also treated with REG as second‐line therapy.

**Table 1:** Characteristics of patients without a reduced lenvatinib dose starting and with observation period more than 2 wk

	All (n = 77)	TKI naïve (n = 33)	TKI experienced (n = 44)	*P* value
Age (y)	72.0 ± 8.9	74.6 ± 11.2	70.0 ± 5.9	0.040
Gender (male:female)	59:18	26:7	33:11	0.705
BMI (kg/m^2^)	22.2 ± 4.2	22.6 ± 3.4	21.9 ± 4.8	0.439
ECOG PS (0:1:2)	67:8:2	28:4:1	39:4:1	0.634
Etiology (HCV:HBV:alcohol:others)	38:14:12:13	14:6:5:8	24:8:7:5	0.188
AST (IU/L)	57.2 ± 67.7	48.8 ± 26.0	63.6 ± 86.6	0.289
ALT (IU/L)	43.9 ± 50.3	42.7 ± 36.3	44.8 ± 59.2	0.853
Platelets (×10^4^/µL)	14.7 ± 5.5	14.1 ± 5.8	15.1 ± 5.4	0.434
Total bilirubin (mg/dL)	0.8 ± 0.4	0.8 ± 0.3	0.9 ± 0.5	0.732
Albumin (g/dL)	3.6 ± 0.5	3.6 ± 0.5	3.6 ± 0.5	0.498
Prothrombin time (%)	90.6 ± 13.3	89.9 ± 13.5	91.2 ± 13.3	0.673
Child‐Pugh score (5:6:7:8)	42:25:9:1	19:11:3:0	23:14:6:1	0.512
ALBI grade (1:2:3)	26:49:2	11:21:1	15:28:1	0.917
[mALBI grade (1:2a:2b:3)]	[26:19:30:2]	[11:7:14:1]	[15:12:16:1]	0.690
(ALBI score)	(−2.32 ± 0.49)	(−2.36 ± 0.49)	(−2.30 ± 0.49)	0.577
AFP (ng/mL)	3248.2 ± 15406.1	2515.9 ± 8252.5	3797.4 ± 19185.9	0.693
Intrahepatic tumor size (cm)	4.1 ± 4.5	2.8 ± 2.3	5.1 ± 5.5	0.019
Number of intrahepatic tumors (none:single:multiple)	9:4:64	5:3:25	4:1:39	0.326
TNM stage, AJCC/UICC 8th (IB:II:IIIA:IIIB:IVA:IVB)	2:27:6:5:9:28	2:17:1:1:4:8	0:10:5:4:5:20	0.028
TNM stage, LCSGJ 6th (II:III:IV) [IVa:IVb]	8:28:41 [4:37]	8:12:13 [1:12]	0:16:28 [3:25]	0.006
Positive for MVI (Vp1:Vp2:Vp3:Vp4:Vv1:Vv2:Vv3)^a^	17 (22.1%) (2:5:4:3:1:2:4)	2 (6.1%) (0:1:1:0:0:0)	15 (34.1%) (2:4:3:3:1:2:4)	0.005
Positive for EHM (lung:LN: bone:peritoneum:adrenal gland:others)^a^	37 (48.1%) 11:15:9:5:2:2	12 (36.4%) (1:5:1:4:0:2)	25 (56.8%) (10:10:8:1:2:0)	0.107
naïve:recurrence	2:75 (median number of past treatments 5)	2:31	0:44	0.180
Past history of hypertension	33 (42.9%)	9(27.3%)	24 (54.5%)	0.021
Past history of diabetes mellitus	21 (27.3%)	11 (33.3%)	10 (22.7%)	0.316
Past history of SOR and REG	44 (57.1%)	—	11 (14.4%) (REG)	—
Initial dose of LEN (8:12 mg/d)	49:28	22:11	27:17	0.811
Average observation period after starting LEN (d)	79.3 ± 40.7	67.1 ± 41.9	88.4 ± 37.7	0.025

AFP, alpha‐fetoprotein; AJCC/UICC 8th, American Joint Committee on Cancer/Union for International Cancer Control, 8th edition; ALBI grade, albumin‐bilirubin grade; ALT, alanine aminotransferase; AST, aspartate transaminase; BMI, body mass index; ECOG PS, Eastern Cooperative Oncology Group performance status; EHM, extra‐hepatic metastasis; HBV, hepatitis B virus; HCV, hepatitis C virus; LCSGJ 6th, the Liver Cancer Study Group of Japan, 6th edition; LEN, lenvatinib; LN, lymph node; MVI, major venous invasion; REG, regorafenib; SOR, sorafenib; TNM stage, tumor node metastasis stage.

a Overlapping cases.

**Figure 2:**
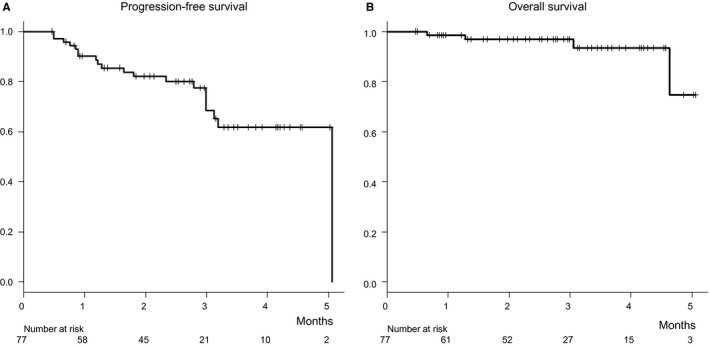
Progression‐free and overall survivals. After 4, 8, and 12 wk of treatment, the progression‐free survival rate (PFSR) was 90.1%, 82.1%, and 80.1%, respectively (A), while overall survival rate (OSR) was 98.6%, 96.9%, and 93.4%, respectively (B)

**Figure 3:**
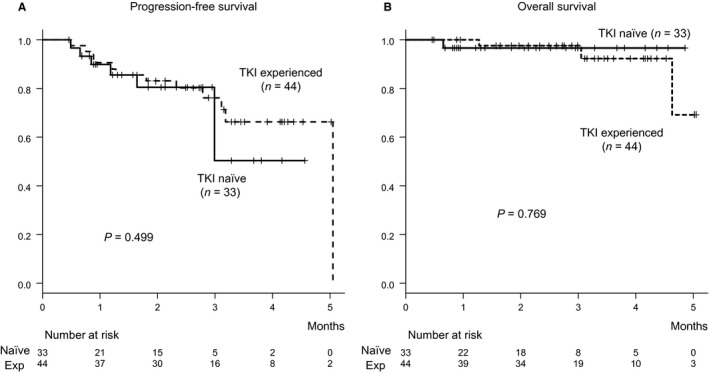
Progression‐free and overall survivals for tyrosine kinase inhibitor‐naïve and TKI‐experienced groups. After 4, 8, and 12 wk of treatment, the progression‐free survival rate (PFSR) and overall survival (OSR) were not significantly different between the tyrosine kinase inhibitor (TKI)‐naïve (solid line) and TKI‐experienced groups (broken line) (PFSR: 89.7%/80.4%/80.4% vs 90.5%/83.0%/80.1%, *P* = 0.499; OSR: 96.7%/96.7%/96.7% vs 100%/97.4%/92.3%, *P* = 0.769)

The first evaluation of the therapeutic effect of LEN using an imaging modality was performed in 52 patients at 4 weeks after introduction of the drug. Complete response (CR) was noted in none, partial response (PR) in 20, stable disease (SD) in 22, and PD in 10 (overall response rate [ORR]: 38.5%, disease control rate [DCR]: 80.8%). In results of CT/MRI imaging of 37 patients evaluated at 12 weeks, CR was noted in 1, PR in 11, SD in 14, and PD in 11 (ORR: 32.4% and DCR: 70.3%). At 4, 8, and 12 weeks, PFSR was 90.1%, 82.1%, and 80.1%, respectively (Figure 2A), while OSR was 98.6%, 96.9%, and 93.4%, respectively (Figure 2B). Although older age, shorter observation period, lower frequency of past history of hypertension, and reduced tumor burden shown by lower TNM stage with both systems were noted in the TKI‐naïve group as compared to the experienced group, there were no significant differences between them in regard to clinical characteristics, especially hepatic reserve function (Child‐Pugh score, ALBI score/grade, mALBI grade) (Table 1). As a result, PFSR and OSR at 4, 8, and 12 weeks were not significantly different between the TKI‐naïve and TKI‐experienced groups (PFSR: naïve group, 89.7%, 80.4%, 80.4% vs experienced group, 90.5%, 83.0%, 80.1%, respectively, *P* = 0.499; OSR: 96.7%, 96.7%, 96.7% vs 100%, 97.4%, 92.3%, respectively, *P* = 0.769) (Figure 3A,B). A significant decline in log10 AFP from the baseline to 4 weeks after introducing LEN was observed in all patients who obtained disease control (PR:SD = 20:22) (baseline vs 4 weeks: 2.047 ± 1.148 vs 1.796 ± 1.179, *P* < 0.001). After exclusion of those with normal AFP level (<10 ng/mL) (n = 13), the significant relative decline remained (baseline vs 4 weeks: 2.662 ± 0.798 vs 2.393 ± 0.946, *P* = 0.002) (n = 29).

**Figure 4:**
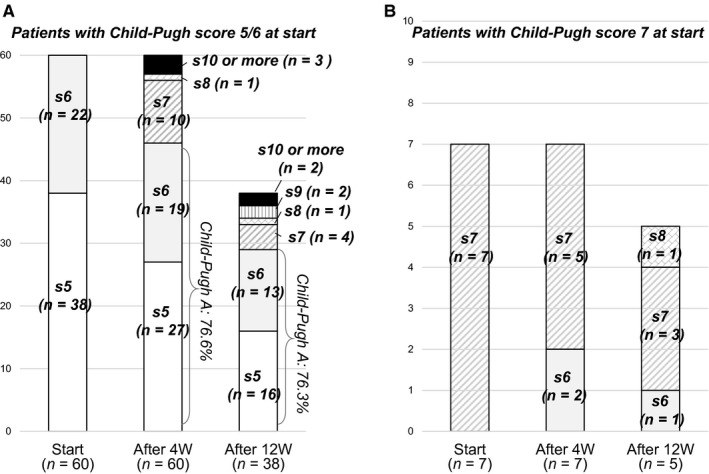
Child‐Pugh score at start, then 4 and 12 weeks after introducing lenvatinib. After starting LEN treatment, Child‐Pugh class A had worsened to Child‐Pugh B or C 23.4% patients at 4 wk (*P* < 0.001) and 23.7% at 12 wk (*P* = 0.002)

**Figure 5:**
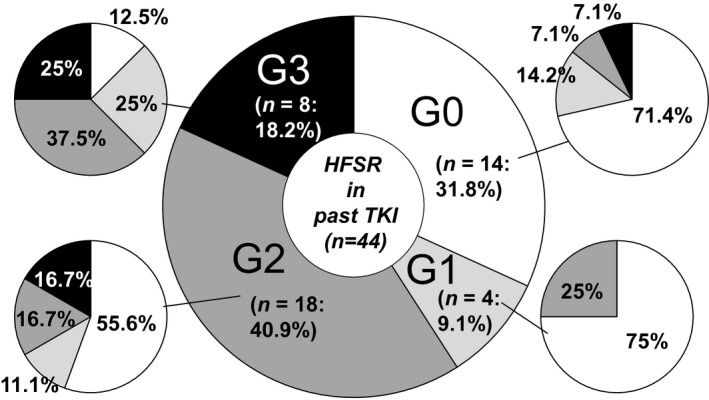
Hand‐foot skin reaction occurrence in tyrosine kinase inhibitor‐experienced group. Of 8 patients who had undergone past tyrosine kinase inhibitor (TKI) treatments and developed a grade 3 hand‐foot skin reaction (HFSR), 87.5% had an HFSR with lenvatinib (LEN) therapy (G3, 2, 1; n = 2, 3, 2, respectively). Furthermore of 18 with a grade 2 HFSR in past TKI treatments, 44.4% had an HFSR with LEN (G3, 2, 1; n = 3, 3, 2, respectively). Of 4 with a grade 1 HFSR in previous TKI treatments, grade 2 occurred in 1 and no HFSR was seen in 3 with LEN treatment. In 14 without an HFSR in past TKI treatments, 28.6% developed HFSR with LEN treatment (G3, 2, 1; n = 3, 3, 2, respectively)

After starting LEN treatment, patients with Child‐Pugh class A had worsened to Child‐Pugh B or C in 23.4% of patients at 4 (*P* < 0.001) and in 23.7% at 12 weeks (*P* = 0.002) (Figure 4). Furthermore, a significant decline in ALBI score from the baseline (−2.32 ± 0.49) was observed at 4 (−2.18 ± 0.54 [n = 67], *P* < 0.001) and 12 (−2.10 ± 0.55 [n = 43], *P* < 0.001) weeks after the start of LEN treatment. AEs that occurred following the start of LEN in these patients are presented in Table 2. HFSR was the most common in the present cohort (all grades: n = 31 [40.3%], grade 3: n = 7 [9.1%]), followed by general fatigue (grades 1/2: n = 26 [33.8%]), appetite loss (all grades: n = 22 [28.6%], grade 3: n = 5 [6.5%]), hoarseness (all grades: n = 17 [22.1%], grade 3: none), and hypothyroidism (all grades: n = 15 [19.5%], grade 3: none). In 3 (3.9%) patients, destructive thyroiditis was observed (grade 2, n = 1; grade 3, n = 2).^25^ Although there was no significant relative change in regard to the level of FT4 (baseline vs 4 weeks: 2.0 ± 4.1 vs 1.1 ± 0.5 ng/dL, *P* = 0.107), TSH level was significantly elevated from the baseline after the first 4 weeks of LEN treatment (baseline vs 4 weeks: 5.9 ± 12.5 vs 15.2 ± 29.7 µIU/mL, *P* = 0.003). Also, the levels of NH3, serum amylase, and estimated glomerular filtration rate (eGFR) did not show significant changes within the first 4 weeks (NH3: 47.7 ± 27.1 vs 47.9 ± 32.7 µg/dL, *P* = 0.548; serum amylase: 88.6 ± 38.4 vs 101.1 ± 19.9 U/L, *P* = 0.809; eGFR: 75.7 ± 19.6 vs 73.0 vs 21.2 mL/min/1.73 m^2^, *P* = 0.279). Among 45 patients who experienced down‐dosing or a pause in LEN treatment due to an AE during the present observation period, LEN was abandoned in 17 (PD, n = 5; AEs, n = 12). The median period to initial dose reduction or pause of LEN was 40 days. Of 8 with an HFSR with a grade of 3 in previous treatments with a TKI, 87.5% also had an HFSR (grade 3, 2, 1; n = 2, 3, 2, respectively) with LEN treatment, while 44.4% of 18 with an HFSR with a grade of 2 during previous TKI treatments had an HFSR with LEN (grade 3, 2, 1; n = 3, 3, 2, respectively) (Figure 5).

**Table 2:** Adverse events of lenvatinib treatment

	All patients (n = 77)			TKI naïve (n = 33)		TKI experienced (n = 44)		*P* value
	G1/2	G3/4	Any grade (%)	G1/2	G3/4	G1/2	G3/4	
HFSR^a^	24	7	31 (40.3)	6/4	1/0	6/8	6/0	0.288
General fatigue^a^	26	—	26 (33.8)	3/6	—	7/8	—	0.727
Appetite loss^a^	17	5	22 (28.6)	1/4	2/0	6/6	3/0	0.451
Hoarseness^a^	17	0	17 (22.1)	9/2	0/—	5/1	0/—	0.104
Hypothyroidism^a^	15	0	15 (19.5)	2/4	0/0	0/9	0/0	0.199
Destructive thyroiditis^a^	1	2	3 (3.9)	0/0	1/0	0/1	1/0	1.000
Hypertension^a^	9	5	14 (18.2)	2/3	2/0	0/4	3/0	0.825
Diarrhea^a^	12	1	13 (16.7)	2/2	0/0	6/2	1/0	0.735
Urine protein^a^	8	3	11 (14.3)	1/3	2/—	2/2	1/—	0.675
Hyperammonemia/hepatic coma^a^	3	2	5 (6.5)	1/1	1/0	0/1	1/0	0.788
Fever within 2 wk^a^	2	1	3 (3.9)	0/0	0/0	1/1	1/0	0.132
Others^a^	24	9	33	2/2	5/1^b^	13/7	3/0	

G, grade; HFSR, hand‐foot skin reaction; TKI, tyrosine kinase inhibitor; —: no setting for the applicable grade in the National Cancer Institute's Common Terminology Criteria for Adverse Events, version 4.0.

a Some events are overlapped.

b Case of hypoglycemia due to appetite loss.

The data of CT imaging findings at introducing LEN were obtained for 51 patients, of whom 18 (35.3%) had MVL. As a sub‐analysis, relative changes in muscle volume at 4 and 12 weeks after introducing LEN were evaluated in 41 and 25 patients, respectively, whose data of CT imaging findings obtained at the start of and approximately 4 and 12 weeks after beginning treatment. In those, the relative change in PI with total psoas muscle area at L3 level in the 41 patients after 4 weeks was −0.210 ± 0.315 cm^2^/m^2^, while that in the 25 patients after 12 weeks was −0.275 ± 0.372 cm^2^/m^2^.

## 4 DISCUSSION

Development of TKI treatment for u‐HCC has improved the prognosis of affected patients. Following the introduction of SOR, global trials of sunitinib,^26^ brivanib,^27^ linifanib,^28^ and erlotinib plus SOR^29^ were conducted, though none of those drugs showed superiority and each of the trials ended in failure. Thus, there was no additional first‐ or second‐line TKI treatment for u‐HCC developed until the introductions of REG^3^, ^4^ and LEN.^6^ Recently, REG was developed as a second‐line option for SOR in patients who met the RESORCE trial criteria,^3^ after which LEN became available as a first‐line oral TKI targeting VEGF receptors 1‐4, PDGF receptor α, RET, and KIT.^6^

In the present study, early therapeutic response with LEN was favorable and patients obtained disease control within 4 weeks, as follow‐up imaging showed a significant decline in log10 AFP from the baseline. Kuzuya et al^30^ have reported that a decreased AFP ratio from the baseline at 4 weeks after introduction of SOR was a predictor of good therapeutic response (PR, SD). Thus, a decline of log10 AFP from the baseline within the first 4 weeks might also have predictive value for therapeutic response in patients receiving LEN treatment. In addition, a previous study noted that fever within 2 weeks after the start of SOR therapy may be useful as a predictor of favorable treatment response in patients with advanced HCC and receiving SOR treatment.^31^ Three of our patients developed a fever within 2 weeks after the start of LEN (grade 1, 2, 3: each 1 patient), in whom therapeutic response was PD in 2 and SD in 1. It would be important to examine the clinical importance of fever in patients receiving LEN therapy as a useful predictor of a favorable treatment response in a future study with a larger number of patients.

Not all patients treated with SOR in clinical practice meet the RESORCE trial criteria; thus, the frequency of u‐HCC patients indicated for REG has been reported to only range from 30.6% to 37.0% of those confirmed by radiological findings to have PD with SOR therapy.^32^, ^33^ Absence of a therapeutic option for second‐line treatment after SOR other than REG in u‐HCC patients who do not meet the RESORCE trial eligibility criteria (tolerability for SOR) is an important clinical issue. Our previous study examined the use of LEN for u‐HCC in both TKI‐naïve and TKI‐experienced patients in real‐world clinical situations in Japan,^7^ as accurate determination of clinical profile differences including detailed therapeutic response and AE occurrence between TKI‐naïve and TKI‐experienced patients has become an urgent clinical issue. In the present results, PFSR and OSR were not significantly different between the TKI‐naïve and TKI‐experienced groups, and similar AE profiles were observed in both. We consider that LEN may have potential to satisfy the unmet need of clinical TKI treatment for u‐HCC. On the other hand, the cohort in this retrospective study included u‐HCC patients classified as Child‐Pugh B and/or with major vein tumor thrombosis. However, indication for LEN treatment in such patients should be determined with caution, because no evidence has been presented showing the safety or effects of LEN in u‐HCC patients classified as Child‐Pugh B and/or with major vein tumor thrombosis.

In the REFLECT trial reported by Kudo,^6^ the frequency of HFSR was less in patients that received SOR (all grades: 26.9% vs 52.4%), whereas all grades of HFSR were observed more frequently in the present analysis (40.3%). Although there was no statistical difference in the frequency of HFSR between our TKI‐naïve and TKI‐experienced groups (33.3% vs 45.5%, *P* = 0.350), past history of high‐grade HFSR might be an important risk factor of it developing during LEN therapy. Thus, HFSR should be kept in mind as a commonly occurring AE in patients with a history of that condition during previous SOR and/or REG treatments. HFSR in association with SOR has been reported as a factor predicting a better therapeutic effect of SOR for HCC.^36^, ^37^ Based on these findings, the relationship between HFSR and therapeutic response with LEN treatment should be analyzed in the future. Nevertheless, countermeasures against HFSR are most important for patients with a past history of HFSR in order to maintain TKI adherence for enhancing the anti‐cancer effect.

In the present cohort, Child‐Pugh class and ALBI score became worse at 4 and 12 weeks after starting LEN treatment. In addition to HFSR, general fatigue and appetite loss were the second and third most frequent AEs seen in the present analysis, and it is thought that LEN and these AEs have direct effects on liver function and nutritional status. To maintain adherence to LEN therapy, it is important not only to monitor and treat as carefully as possible AEs but also to keep in mind to introduce LEN, as well as SOR, as possible as in better liver function, if necessary for LEN treatment.

HCC is often seen in patients with chronic liver disease (CLD). Additionally, MVL is not rare in affected patients^14^ and its frequency was shown to increase in association with the progression of CLD stage.^38^ Moreover, it has been reported that MVL is a more significant prognostic factor than portal hypertension in liver cirrhosis (LC) patients (Child‐Pugh class A/B),^39^ and also an important prognostic factor following not only surgical resection^40^ but also SOR treatment^41^ in patients with HCC. Recently, a meta‐analysis study reported that MVL was a prognostic factor regardless of therapeutic modality given for HCC by Chang et al.^42^ Furthermore, in addition to LC and HCC, muscle volume has been reported to be an important prognostic factor in patients receiving treatments for other types of cancers.^43^ In the present study, a fair percentage of our patients (35.3%) had MVL and a relative decline in muscle volume at 4 and 12 weeks after starting LEN was observed in patients who had data of CT imaging findings available, similar to our past study of patients treated with SOR.^41^ It will be important to analyze the relationships among therapeutic response, OSR, and muscle volume in patients undergoing LEN therapy in a future study. Although some trials of intervention with nutrition and exercise CLD patients have been reported,^44^, ^45^ effective intervention methods have yet to be reported. Establishment of strategies for preventing and improving muscle wasting in CLD patients with and without HCC is needed for improving prognosis.

The present study has some limitations, including its retrospective nature. Additionally, though this was a multicenter study, the number of analyzed patients was not large and the observation period was limited. In a future study, we hope to examine the relationship between prognosis and clinical features including AEs and muscle volume with a larger number of patients receiving LEN therapy.

In summary, regardless of previous TKI treatments, therapeutic response and AE occurrence following introduction of LEN treatments were similar. We consider that LEN might complement the unmet need for TKI therapy for patients with u‐HCC.

## CONFLICT OF INTEREST

None declared.

## References

[1] Llovet JM , Ricci S , Mazzaferro V , et al. Sorafenib in advanced hepatocellular carcinoma. N Engl J Med. 2008;359:378‐390. 18650514 10.1056/NEJMoa0708857

[2] Cheng AL , Kang YK , Chen Z , et al. Efficacy and safety of sorafenib in patients in the Asia‐Pacific region with advanced hepatocellular carcinoma: a phase III randomised, double‐blind, placebo‐controlled trial. Lancet Oncol. 2009;10:25‐34. 19095497 10.1016/S1470-2045(08)70285-7

[3] Bruix J , Qin S , Merle P , et al. Regorafenib for patients with hepatocellular carcinoma who progressed on sorafenib treatment (RESORCE): a randomised, double‐blind, placebo‐controlled, phase 3 trial. Lancet. 2017;389:56‐66. 27932229 10.1016/S0140-6736(16)32453-9

[4] Finn RS , Merle P , Granito A , et al. Outcomes of sequential treatment with sorafenib followed by regorafenib for HCC: additional analyses from the phase 3 RESORCE trial. J Hepatol. 2018;69:353‐358. 29704513 10.1016/j.jhep.2018.04.010

[5] Ikeda K , Kudo M , Kawazoe S , et al. Phase 2 study of lenvatinib in patients with advanced hepatocellular carcinoma. J Gastroenterol. 2017;52:512‐519. 27704266 10.1007/s00535-016-1263-4 PMC5357473

[6] Kudo M , Finn RS , Qin S , et al. Lenvatinib versus sorafenib in first‐line treatment of patients with unresectable hepatocellular carcinoma: a randomised phase 3 non‐inferiority trial. Lancet. 2018;391:1163‐1173. 29433850 10.1016/S0140-6736(18)30207-1

[7] Hiraoka A , Kumada T , Kariyama K , Takaguchi K , Itobayashi E , Shimada N . Therapeutic potential of lenvatinib for unresectable hepatocellular carcinoma in clinical practice: multicenter analysis. Hepatol Res. 2018 10.1111/hepr.13243 [Epub ahead of print]. 30144256

[8] Pugh RN , Murray‐Lyon IM , Dawson JL , Pietroni MC , Williams R . Transection of the oesophagus for bleeding oesophageal varices. Br J Surg. 1973;60:646‐649. 4541913 10.1002/bjs.1800600817

[9] Johnson PJ , Berhane S , Kagebayashi C , et al. Assessment of liver function in patients with hepatocellular carcinoma: a new evidence‐based approach‐the ALBI grade. J Clin Oncol. 2015;33:550‐558. 25512453 10.1200/JCO.2014.57.9151 PMC4322258

[10] Hiraoka A , Kumada T , Michitaka K , et al. Usefulness of albumin‐bilirubin grade for evaluation of prognosis of 2584 Japanese patients with hepatocellular carcinoma. J Gastroenterol Hepatol. 2016;31:1031‐1036. 26647219 10.1111/jgh.13250

[11] Hiraoka A , Kumada T , Kudo M , et al. Albumin‐Bilirubin (ALBI) grade as part of the Evidence‐Based Clinical Practice Guideline for HCC of the Japan Society of Hepatology: a comparison with the liver damage and child‐pugh classifications. Liver Cancer. 2017;6:204‐215. 28626732 10.1159/000452846 PMC5473065

[12] Hiraoka A , Michitaka K , Kumada T , et al. Validation and potential of albumin‐bilirubin grade and prognostication in a nationwide survey of 46,681 hepatocellular carcinoma patients in Japan: the need for a more detailed evaluation of hepatic function. Liver Cancer. 2017;6:325‐336. 29234636 10.1159/000479984 PMC5704689

[13] Hiraoka A , Kumada T , Tsuji K , et al. Validation of modified ALBI grade for more detailed assessing hepatic function of hepatocellular carcinoma ‐ multicenter analysis. Liver Cancer. 2018 10.1159/000488778 [Epub ahead of print]. PMC6465715 31019902

[14] Hiraoka A , Aibiki T , Okudaira T , et al. Muscle atrophy as pre‐sarcopenia in Japanese patients with chronic liver disease: computed tomography is useful for evaluation. J Gastroenterol. 2015;50:1206‐1213. 25820219 10.1007/s00535-015-1068-x PMC4673094

[15] Bruix J , Sherman M . Management of hepatocellular carcinoma. Hepatology. 2005;42:1208‐1236. 16250051 10.1002/hep.20933

[16] Di Martino M , Marin D , Guerrisi A , et al. Intraindividual comparison of gadoxetate disodium‐enhanced MR imaging and 64‐section multidetector CT in the Detection of hepatocellular carcinoma in patients with cirrhosis. Radiology. 2010;256:806‐816. 20720069 10.1148/radiol.10091334

[17] Sano K , Ichikawa T , Motosugi U , et al. Imaging study of early hepatocellular carcinoma: usefulness of gadoxetic acid‐enhanced MR imaging. Radiology. 2011;261:834‐844. 21998047 10.1148/radiol.11101840

[18] Hiraoka A , Ichiryu M , Tazuya N , et al. Clinical translation in the treatment of hepatocellular carcinoma following the introduction of contrast‐enhanced ultrasonography with Sonazoid. Oncol Lett. 2010;1:57‐61. 22966256 10.3892/ol_00000010 PMC3436383

[19] Hiraoka A , Hiasa Y , Onji M , Michitaka K . New contrast enhanced ultrasonography agent: impact of Sonazoid on radiofrequency ablation. J Gastroenterol Hepatol. 2011;26:616‐618. 21418298 10.1111/j.1440-1746.2011.06678.x

[20] The Liver Cancer Study Group of Japan . The General Rules for the Clinical and Pathological Study of Primary Liver Cancer, 6th edn Kanehara: Tokyo; 2015:26.

[21] National Cancer Institute , Protocol Development Cancer Therapy . https://ctep.cancer.gov/protocolDevelopment/electronic_applications/ctc.htm-ctc_40. Accessed May 31, 2018.

[22] Eisenhauer EA , Therasse P , Bogaerts J , et al. New response evaluation criteria in solid tumours: revised RECIST guideline (version 1.1). Eur J Cancer. 2009;45:228‐247. 19097774 10.1016/j.ejca.2008.10.026

[23] Lencioni R , Llovet JM . Modified RECIST (mRECIST) assessment for hepatocellular carcinoma. Semin Liver Dis. 2010;30:52‐60. 20175033 10.1055/s-0030-1247132 PMC12268942

[24] Kanda Y . Investigation of the freely available easy‐to‐use software ‘EZR’ for medical statistics. Bone Marrow Transplant. 2013;48:452‐458. 23208313 10.1038/bmt.2012.244 PMC3590441

[25] Hirooka M , Ochi H , Hiraoka A , et al. Destructive thyroiditis induced by lenvatinib in three patients with hepatocellular carcinoma. Internal Med. 2018 10.2169/internalmedicine.1874-18 [Epub ahead of print]. PMC6465025 30333428

[26] Cheng AL , Kang YK , Lin DY , et al. Sunitinib versus sorafenib in advanced hepatocellular cancer: results of a randomized phase III trial. J Clin Oncol. 2013;31:4067‐4075. 24081937 10.1200/JCO.2012.45.8372

[27] Johnson PJ , Qin S , Park JW , et al. Brivanib versus sorafenib as first‐line therapy in patients with unresectable, advanced hepatocellular carcinoma: results from the randomized phase III BRISK‐FL study. J Clin Oncol. 2013;31:3517‐3524. 23980084 10.1200/JCO.2012.48.4410

[28] Cainap C , Qin S , Huang WT , et al. Linifanib versus Sorafenib in patients with advanced hepatocellular carcinoma: results of a randomized phase III trial. J Clin Oncol. 2015;33:172‐179. 25488963 10.1200/JCO.2013.54.3298 PMC4279237

[29] Zhu AX , Rosmorduc O , Evans TR , et al. SEARCH: a phase III, randomized, double‐blind, placebo‐controlled trial of sorafenib plus erlotinib in patients with advanced hepatocellular carcinoma. J Clin Oncol. 2015;33:559‐566. 25547503 10.1200/JCO.2013.53.7746

[30] Kuzuya T , Asahina Y , Tsuchiya K , et al. Early decrease in alpha‐fetoprotein, but not des‐gamma‐carboxy prothrombin, predicts sorafenib efficacy in patients with advanced hepatocellular carcinoma. Oncology. 2011;81:251‐258. 22116493 10.1159/000334454

[31] Kuzuya T , Ishigami M , Ishizu Y , et al. Fever within 2 weeks of Sorafenib therapy predicts favorable treatment efficacy in patients with advanced hepatocellular carcinoma. Oncology. 2016;91:261‐266. 27622905 10.1159/000449000

[32] Kuzuya T , Ishigami M , Ishizu Y , et al. Prognostic factors associated with postprogression survival in advanced hepatocellular carcinoma patients treated with Sorafenib not eligible for second‐line regorafenib treatment. Oncology. 2018;95:91‐99. 29723866 10.1159/000488453

[33] Shinsuke U , Kawaoka T , Aikata H , et al. Clinical outcomes of sorafenib treatment failure for advanced hepatocellular carcinoma and candidates for regorafenib treatment in real‐world practice. Hepatol Res. 2018. 10.1111/hepr.13180 29682855

[34] Terashima T , Yamashita T , Sunagozaka H , et al. Analysis of the liver functional reserve of patients with advanced hepatocellular carcinoma undergoing sorafenib treatment: prospects for regorafenib therapy. Hepatol Res. 2018;48:956‐966. 29845710 10.1111/hepr.13196

[35] Ogasawara S , Chiba T , Ooka Y , et al. Characteristics of patients with sorafenib‐treated advanced hepatocellular carcinoma eligible for second‐line treatment. Invest New Drugs. 2018;36:332‐339. 28891038 10.1007/s10637-017-0507-3

[36] Ogawa C , Morita M , Omura A , et al. Hand‐foot syndrome and post‐progression treatment are the good predictors of better survival in advanced hepatocellular carcinoma treated with sorafenib: a multicenter study. Oncology. 2017;93(Suppl 1):113‐119. 29258090 10.1159/000481241

[37] Wang P , Tan G , Zhu M , Li W , Zhai B , Sun X . Hand‐foot skin reaction is a beneficial indicator of sorafenib therapy for patients with hepatocellular carcinoma: a systemic review and meta‐analysis. Expert Rev Gastroenterol Hepatol. 2018;12:1‐8. 28847184 10.1080/17474124.2017.1373018

[38] Hiraoka A , Michitaka K , Ueki H , et al. Sarcopenia and two types of presarcopenia in Japanese patients with chronic liver disease. Eur J Gastroenterol Hepatol. 2016;28:940‐947. 27232361 10.1097/MEG.0000000000000661

[39] Hiraoka A , Kitahata S , Izumoto H , et al. Muscle volume loss a prognostic factor for death in liver cirrhosis patients and special relationship to portal hypertension. Hepatol Res. 2018;48:E354‐E359. 28940597 10.1111/hepr.12984

[40] Hiraoka A , Otsuka Y , Kawasaki H , et al. Impact of muscle volume and muscle function decline in patients undergoing surgical resection for hepatocellular carcinoma. J Gastroenterol Hepatol. 2018;33:1271‐1276. 29193248 10.1111/jgh.14058

[41] Hiraoka A , Hirooka M , Koizumi Y , et al. Muscle volume loss as a prognostic marker in hepatocellular carcinoma patients treated with sorafenib. Hepatol Res. 2017;47:558‐565. 27480045 10.1111/hepr.12780

[42] Chang KV , Chen JD , Wu WT , Huang KC , Hsu CT , Han DS . Association between loss of skeletal muscle mass and mortality and tumor recurrence in hepatocellular carcinoma: a systematic review and meta‐analysis. Liver Cancer. 2018;7:90‐103. 29662836 10.1159/000484950 PMC5892377

[43] Martin L , Birdsell L , Macdonald N , et al. Cancer cachexia in the age of obesity: skeletal muscle depletion is a powerful prognostic factor, independent of body mass index. J Clin Oncol. 2013;31:1539‐1547. 23530101 10.1200/JCO.2012.45.2722

[44] Hiraoka A , Michitaka K , Kiguchi D , et al. Efficacy of branched‐chain amino acid supplementation and walking exercise for preventing sarcopenia in patients with liver cirrhosis. Eur J Gastroenterol Hepatol. 2017;29:1416‐1423. 29016470 10.1097/MEG.0000000000000986

[45] Koya S , Kawaguchi T , Hashida R , et al. Effects of in‐hospital exercise on liver function, physical ability, and muscle mass during treatment of hepatoma in patients with chronic liver disease. Hepatol Res. 2017;47:E22‐E34. 27062043 10.1111/hepr.12718

